# Electrochemical Impedance Spectroscopy Investigation of the SEI Formed on Lithium Metal Anodes

**DOI:** 10.1021/acselectrochem.5c00388

**Published:** 2025-11-24

**Authors:** Lorenz F. Olbrich, Nicolò Pianta, Ben Jagger, Yiming Xu, Manav Kakkanat, Federico Scarpioni, Christopher Allen, Fabio La Mantia, Riccardo Ruffo, Mauro Pasta

**Affiliations:** † Department of Materials, 6396 University of Oxford, Oxford OX1 3PH, United Kingdom; ‡ Department of Materials Science, 9305 University Milano—Bicocca, Via Roberto Cozzi 55, Milan, 20125, Italy; § 28456 Fraunhofer Institute for Manufacturing Technology and Advanced Materials IFAM, Wiener Strasse 12, Bremen 28359, Germany; ∥ Electron Physical Science Imaging Centre, 120796 Diamond Light Source Ltd., Didcot OX11 0DE, United Kingdom; ⊥ 9168 Universität Bremen, Energiespeicher- und Energiewandlersysteme, Bibliothekstrasse 1, Bremen 28359, Germany

**Keywords:** lithium metal anode, solid electrolyte interphase, electrochemical impedance spectroscopy, equivalent circuit modeling, statistical analysis, degrees of freedom

## Abstract

Electrochemical impedance spectroscopy (EIS) is widely used to probe the solid electrolyte interphase (SEI) under realistic conditions, without causing damage to its structure. However, the models and experimental conditions often raise concerns about the reliability of the results. In this work, we present an extensive EIS study of lithium metal in the model electrolyte lithium bis­(fluorosulfonyl)­imide in tetraglyme, analyzing the system at equilibrium as a function of time, temperature, and salt concentration using a setup designed to minimize artifacts. We apply information theory to determine the number of independent degrees of freedom and constrain the number of Voigt elements used in fitting. Our analysis reveals strong correlations among processes, warranting caution when assigning physical meaning. X-ray photoelectron spectroscopy and 4D-scanning transmission electron microscopy measurements are used to support the interpretation and provide complementary insights into the chemical nature of the interphase. The unique and extensive dataset we have collected, comprising over 12000 highly reproducible impedance spectra, will serve as a valuable resource to the community for further analysis and for supporting additional modeling and experimental efforts.

## Graphical abstract

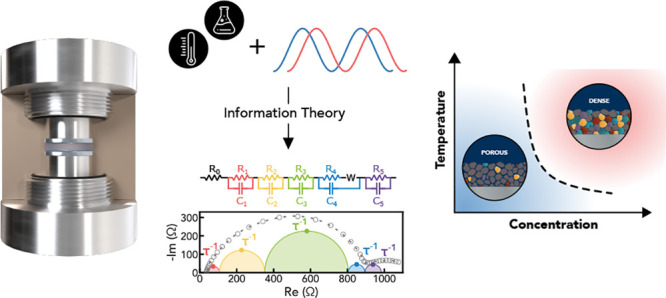

## Introduction

Lithium metal is considered the ultimate negative electrode in rechargeable lithium batteries due to its high specific capacity and lowest electrode potential.^1,2^ However, this low potential causes the spontaneous decomposition of any electrolyte in contact with lithium, resulting in the formation of a solid electrolyte interphase (SEI), first defined by Peled in 1979.^3^ The properties of the SEI directly influence the plating and stripping behavior of lithium metal.^4^ Establishing a connection between SEI characteristics and electrolyte properties is therefore central to the discovery and optimization of new electrolyte formulations.

Characterizing the SEI remains challenging due to its nanoscale thickness and chemical sensitivity, which complicate both sample preparation and analysis, especially when using techniques prone to damaging the sample.^4^ Electrochemical impedance spectroscopy (EIS) offers a nondestructive, operando approach to probe the SEI, providing insight into its structure and evolution under realistic conditions. Accordingly, EIS has been extensively employed to study the SEI, and numerous models have been proposed to extract physical meaning from impedance data.

The most common approach involves fitting the impedance response using Voigt elements (i.e., a resistor in parallel with a capacitor), often modified with constant phase elements (CPEs), both for lithium metal^5–7^ or for other surfaces.^8–11^ These models are typically constructed based on hypothesized physical interpretations of the SEI’s internal structure. The advantage of using a constant phase element (CPE) lies in its ability to provide better fits to impedance data when a distribution of time constants is present. It is important to emphasize, however, that the CPE does not have a direct correspondence to physical properties such as the thickness or dielectric constant of a film. Consequently, relating the CPE-derived distribution of time constants to its physical origin (e.g., electrode porosity, surface inhomogeneity, or compositional variations) is neither straightforward nor unambiguous.^12^

Over the years, alternative models have been proposed to describe the impedance of the SEI that do not rely on the use of a CPE. In 1993, Aurbach et al. proposed a multilayer model in which the SEI is composed of several layers with distinct compositions and thicknesses.^13–16^ This model was represented as a series of Voigt elements, with each element corresponding to a specific layer. The authors suggested that Voigt elements with larger time constants represent the outer porous layers of the SEI, whereas those with smaller time constants correspond to the compact inner layer.^16^

In 1997, Peled et al. introduced a model based on the “mosaic model” proposed in their original work.^3,17^ This model accounts for both grain boundary resistance and charge transfer through individual SEI particles. While the full model is relatively complex, the authors presented a simplified version that closely resembles Aurbach’s multilayer model but offers a different physical interpretation of the circuit elements.

Variations on the multilayer approach remain widely used, with most implementations consisting of multiple Voigt elements representing the SEI, alongside additional components to capture specific features such as electron transfer resistance^18^ or native passivation layers.^19^

An alternative strategy was introduced by Drvarič Talian et al.,^20^ who modeled SEI impedance using a transmission line model (TLM),^21,22^ featuring two parallel ion-transport pathways—one for cations and one for anions—connected by a bridging capacitor. The porous region is represented by the transmission line itself, while the compact inner layer is described by terminal resistance and capacitance elements. This model requires fewer assumptions about layering, offering a different perspective on SEI impedance.

Despite broad similarities, the diversity in circuit structures and parameter interpretations across the literature reflects both the inherent complexity of the SEI and the difficulty in reaching consensus. Inconsistencies in experimental conditions—including variations in cell geometry, equilibrium versus dynamic states, and electrolyte composition—further contribute to the challenge of interpreting impedance data consistently.

In this work, we use an information-theory-guided fitting approach to EIS to investigate symmetric lithium metal cells containing lithium bis­(fluorosulfonyl)­imide (LiFSI) in tetraglyme (G4). To support the interpretation of the EIS data, we complement our analysis with X-ray photoelectron spectroscopy (XPS) and four-dimensional scanning transmission electron microscopy (4D-STEM). The electrolyte was selected due to LiFSI’s known ability to promote stable SEI formation, the high reductive stability of G4, and the extensive characterization of the transport and thermodynamic properties of LiFSI–G4 across a range of concentrations and temperatures.^23–25^

## Experimental Section

### Cell Design

Cell geometry is a critical factor in minimizing artifacts that may distort the impedance response and result in misinterpretation. Coin cells are among the most commonly used formats in the literature due to their ease of assembly. Figure 1a presents an EIS spectrum collected using a coin cell setup with a glass fiber separator and a 12 mm diameter lithium electrode. The two semicircles observed in the Nyquist plot are commonly reported in the literature and are often attributed to distinct features of the SEI. However, by comparing the impedance behavior across different cell formats, Drvarič Talian et al. demonstrated that this response actually arises in cells with an undersized lithium electrode. They attribute it to the electrolyte being in contact with both lithium—where charge transfer readily occurs—and with the exposed current collector, which effectively behaves as a blocking electrode.^26^

**Figure 1:**
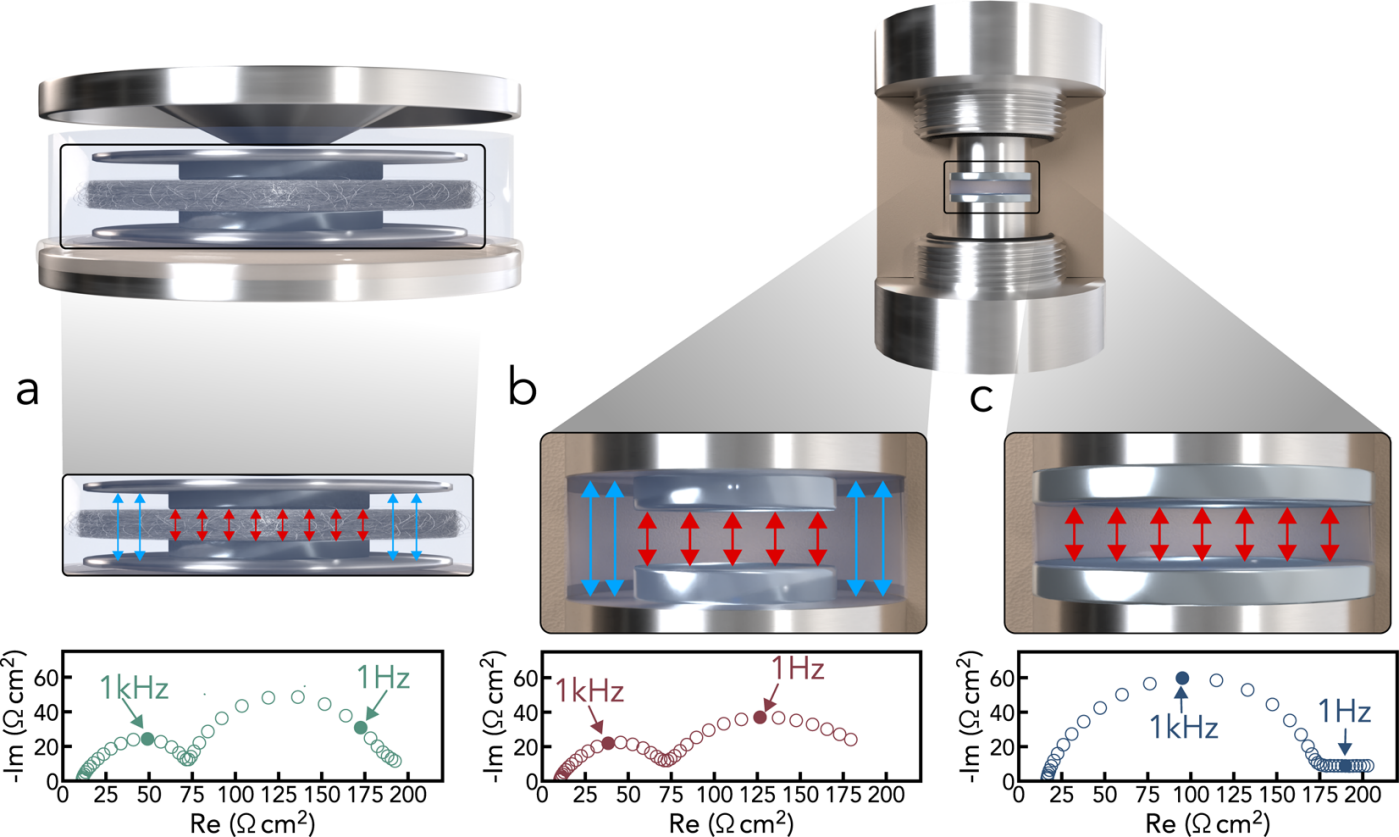
Comparison between different cell geometries. Schematic representation of a coin cell (a) and custom-made cells with partially (b) or fully (c) covered current collectors, along with their respective representative impedance spectra.

Panels b and c of Figure 1 show spectra measured using a custom-made cell consisting of a polyether ether ketone (PEEK) annular cylinder (internal diameter: 8 mm), with two stainless steel pistons serving as current collectors, separated by a 1 mm gap. In this configuration, lithium electrodes with diameters of 4 and 8 mm were used in Figure 1b and Figure 1c, respectively. The data clearly confirm that an undersized lithium electrode leads to the appearance of a second semicircle in the impedance spectra. All the subsequent experiments were therefore conducted in the custom-made PEEK cell shown in Figure 1c.

EIS spectra were collected using different concentrations of LiFSI in G4 (0.25, 1, and 2 m) at various temperatures (20, 30, 40, and 50 °C) under open-circuit conditions. For each set of conditions, a minimum of three cells were measured. Spectra were continuously collected for 10 h immediately after assembly, resulting in a dataset of over 12000 EIS spectra. The full dataset is provided as a Zenodo repository (https://zenodo.org/uploads/15881721).

### Statistical Determination of the Degrees of Freedom

Although most models proposed in the literature are modified versions of the multilayer model introduced by Aurbach et al., a key distinction among them lies in the number of parameters used to fit the data. These range from as few as two^5^ to as many as 14.^16^ In this study, we employed a statistical method to determine the optimal number of parameters (e.g., resistances, capacitances) required to adequately capture the information embedded in the impedance spectra.

The degrees of freedom (DOF) of the system in the probed frequency range are the number of coefficients of the transfer function that define the input/output relation, written as

1 $$H\left(s,\theta_{0}\right)=\frac{1+a_{1}s+a_{2}s^{2}+...+a_{n}s^{n}}{b_{0}+b_{1}s+b_{2}s^{2}+...+b_{n}s^{n}}$$

where *s* is complex and *θ*_0_ = [*b*_0_, *a*_1_, *b*_1_, ..., *a*_*n*_, *b*_*n*_] are the coefficients of the polynomials. Choosing $s=\sqrt{j\omega }$ makes the function applicable for the case of the impedance with semi-infinite Warburg’s diffusion. Such a transfer function is mathematically equivalent to simple equivalent circuit models, including the Voigt and Randles circuits.^27,28^

The appropriate order *n* for the polynomial was determined by fitting all of the datasets with a value of *n* varying from 2 to 10 (equivalent to 3–21 coefficients) and assessing the best one by the use of a model selection criterion. Specifically, we selected the Akaike Information Criterion (AIC) in its form proposed by Ingdal et al. for immittance spectroscopy,^29^ that balances the goodness of fit with model complexity to avoid overfitting. Lower AIC values indicate a good representation of the data with a model of fewest parameters.^30,31^ To corroborate the suggestion of the model selection criterion, we also evaluated the rank of the Jacobian for the linearized function (first-order Taylor approximation):

2 $$Z\left(s,\theta \right)=Z\left(s,\theta_{0}\right)+J\left(\theta_{0}\right)\left(\theta -\theta_{0}\right)$$

where ***J*** is the Jacobian matrix, i.e., the matrix of partial derivatives of ***Z*** with respect to each parameter, evaluated at *θ*_0_. The rank of ***J*** corresponds to the number of linearly independent vectors needed to describe the system. If the rank of ***J*** is lower than the number of parameters in *θ*, some parameters are redundant, and their presence doesn’t affect the value of ***Z*** (*s*, *θ*).

The results of the analysis are shown in Figure 2, where the average rank­(***J***), AIC, and *R* ^2^ for the entire dataset (all concentrations, temperatures, and cell repetitions) are plotted as a function of the transfer function order *n*. The AIC reaches its minimum between *n* = 5 and *n* = 9 (corresponding to 11–14 parameters), while rank­(***J***) indicates that 14 is the number of independent parameters at *n* = 7. From this, we deduce that the system has 14 degrees of freedom. Notably, *R* ^2^ exceeds 0.999 already at *n* = 4, showing that reliance on *R* ^2^ alone would misleadingly suggest that no additional elements are needed. In contrast, the combined use of AIC and rank­(***J***) provides a statistically rigorous criterion for determining the justified number of degrees of freedom.

**Figure 2:**
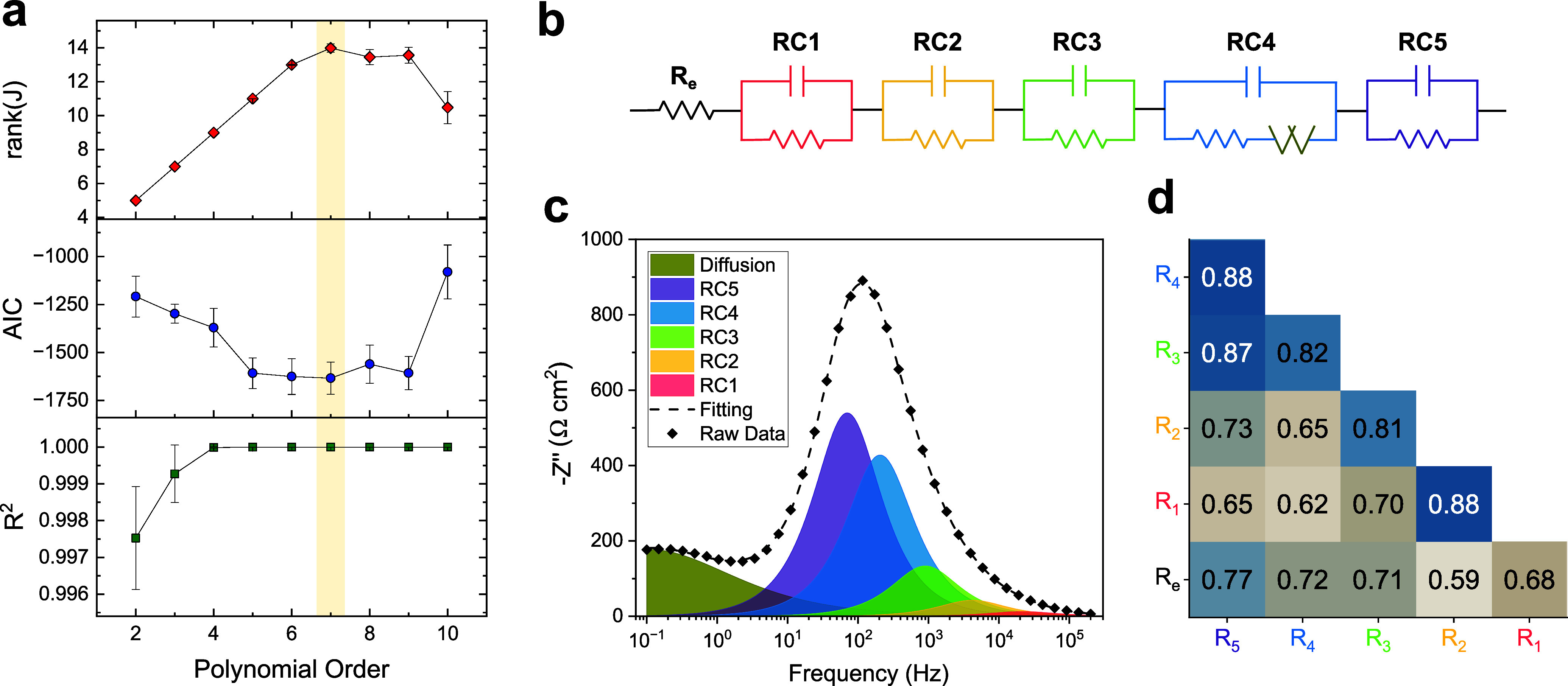
Degrees of freedom estimation and impedance fitting. (a) Average DOF, AlC, and *R* ^2^ as a function of the polynomial order (see eq 1) used to fit the spectra for degrees-of-freedom estimation. The highlighted region marks the range of orders where AIC is minimized, which was used to identify the justified number of elements in the fitting. (b) Equivalent circuit representation of the semi-empirical model, with Voigt elements sorted by increasing time constant *τ*_*i*_ = *R*_*i*_ *C*_*i*_. (c) Results of the fitting for an example impedance in the form of −*Z* vs frequency (data collected at 20 °C, 0.25 m concentration of electrolyte, 5 h from cell assembly). The contribution of the diffusion element has been separated from that of the Randles circuit that contains it for clarity. (d) Average PCC evaluated between the time series of each resistance at different electrolyte concentrations and temperatures.

## Results and Discussions

### Equivalent Model Circuit: Fitting and Analysis

For the physical evaluation of the impedance of the SEI, we fit the data with an equivalent circuit model made of a series of Voigt elements for a total of 14 elements as estimated in the preceding step. To capture the low-frequency response, we insert a distorted bounded-transmissive diffusion element whose characteristic diffusion time constant matches that of the fourth RC branch, ordered from fastest to slowest.^16^ The final circuit is reported in Figure 2b, while Figure 2c highlights the contribution of each element to the total time-constants distribution.

The operating conditions we selected (i.e., absence of separator, fully covered current collector, and open-circuit voltage) allow us to attribute the five RC elements solely to the impedance response of the SEI.^20,32^ The contribution of charge transfer was deliberately excluded, as it has previously been shown to be negligible under these conditions.^18^

Figure 2d shows a heatmap of the average Pearson correlation coefficients (PCCs) between the time-dependent resistances obtained from fitting the raw data. PCCs quantify the strength and direction of a linear relationship between two variables.^33^ The PCC values are uniformly high, ranging from 0.6 to 0.9, indicating strong correlations among the resistances. While this does not necessarily imply that the fitted elements are indistinguishable—since spurious correlations could also produce this effect—it does serve as an initial indication that the elements may reflect a common, distributed phenomenon. With this in mind, we proceeded to investigate their dependence on temperature, concentration, and time.

### Time Constants Analysis

Changes in salt concentration and temperature are known to influence both the composition and nanostructure of the SEI, as well as their evolution over time.^34^ To attempt to decouple these effects, we focused our analysis on trends in time constants (*τ*) of the Voigt elements, rather than on resistance (*R*) and capacitance (*C*) individually. This is because *R* and *C* are sensitive to both composition and morphology, as shown by the following expressions:

3 $$R=\rho \frac{l}{A}$$

4 $$C=\varepsilon \frac{A}{l}$$

where *ρ* and *ε* are the resistivity and permittivity of the SEI, respectively—quantities strongly influenced by compositional changes—while *l* and *A* denote the SEI’s thickness and interfacial surface area, which are more closely tied to its nanostructure. In contrast, the time constant *τ* for a given process, defined as

5 $$\tau_{i}=R_{i}C_{i}=\rho_{i}\varepsilon_{i}$$

depends only on *ρ* and *ε* and is therefore largely insensitive to morphological variations. We analyzed the time constants *τ*_*i*_ of each Voigt element in the model as a function of salt concentration, temperature, and time to probe the compositional evolution of the SEI. The time constants after *stabilization* (defined here as the average over the final hour of measurement; see time-dependence discussion below) are plotted as a function of temperature in Figure 3a–c, for electrolyte concentrations of 0.25, 1, and 2 m, respectively.

**Figure 3:**
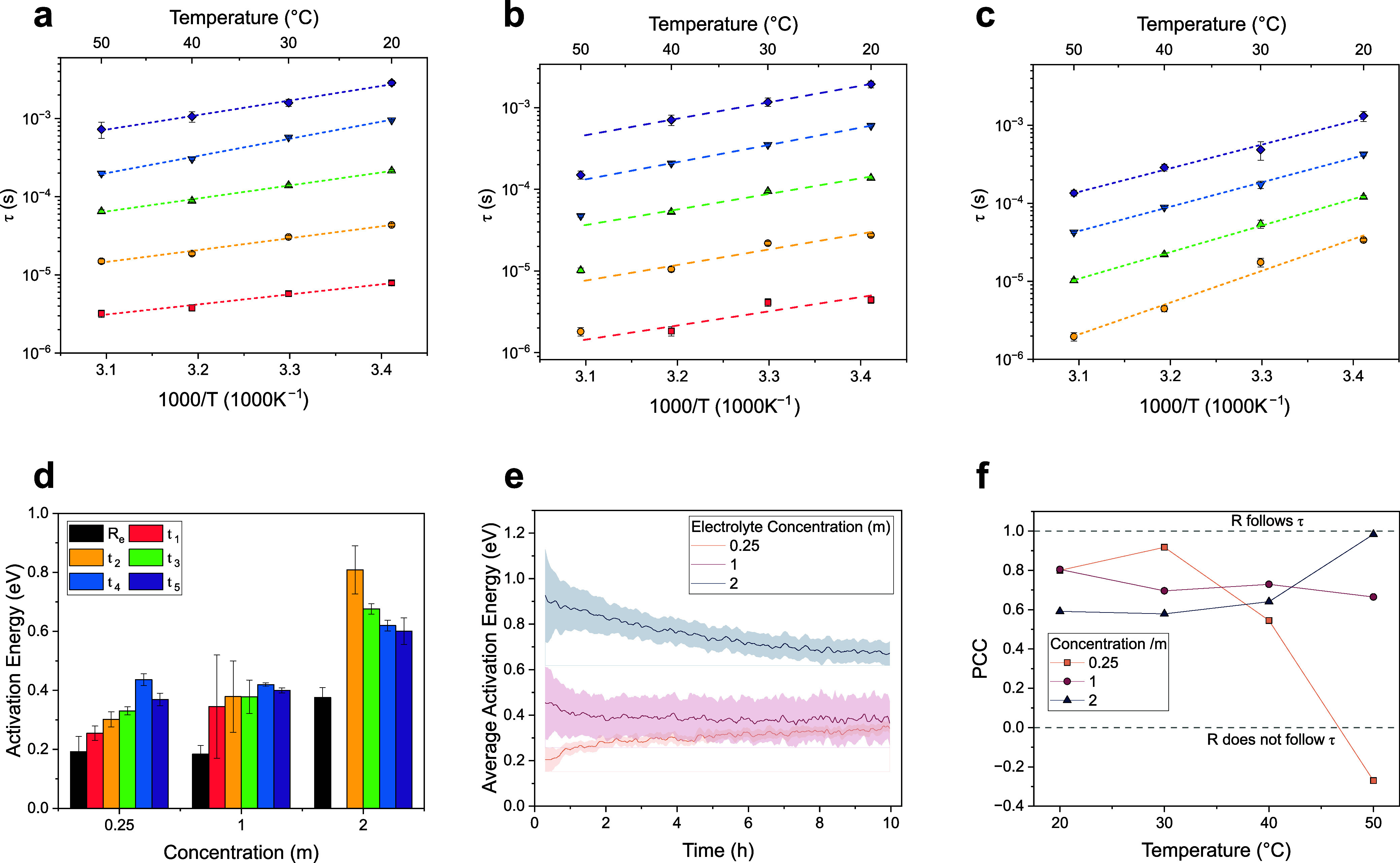
Time constants and activation energy analysis. Time constants of Voigt elements after stabilization for electrolyte concentrations of (a) 0.25, (b) 1, and (c) 2 m. (d) Activation energies of *τ*_*i*_ after stabilization. (e) Time series of the average activation energies. (f) Pearson correlation coefficient between the time series of *R*_*i*_ and the corresponding *τ*_*i*_ at different concentrations and temperatures. For the 1 m dataset, the analysis was performed over the 20–40 °C temperature range.

At salt concentrations of 0.25 m, and at 1 m up to 40 °C, all time constants *τ*_*i*_ exhibit a linear dependence on *T* ^–1^, as shown in Figure 3a,b. This is indicative of Arrhenius-like behavior, supported by high coefficients of determination (*R* ^2^ > 0.95). Activation energies were calculated ranging from 0.2 to 0.4 eV for all time constants (Figure 3c,d). These results suggest that the underlying electrochemical processes—and, by extension, the SEI composition—remain largely consistent across this temperature range and between the two electrolyte concentrations.

A deviation from this trend is observed at 1 m and 50 °C, where the time constants fall significantly below the linear relationship observed at other temperatures. In particular, *τ*_1_ decreases beyond the measurable frequency range, suggesting a change in SEI composition toward more conductive components.

When the electrolyte concentration is increased to 2 m (Figure 3c), compositional effects on the SEI become evident at all temperatures. First, the *τ*_*i*_ values show greater deviations from linearity with respect to *T* ^–1^ (*R* ^2^ ≈ 0.92), suggesting that the SEI composition varies with temperature. Furthermore, the activation energies increase significantly, ranging from 0.6 to 0.8 eV.

The variation of *τ* with time is also a function of concentration and temperature (Supporting Information Figure S1). Overall, *τ* tends to asymptotically approach a stable value over time, which motivates the definition of the stabilized *τ* used above. This occurs rapidly—within less than 2 h—at electrolyte concentrations between 0.25 and 1 m, but is significantly slower at higher concentrations, often extending throughout the entire measurement period (10 h). Notably, in the 1 m, 50 °C condition, all time constants initially increase and then gradually decrease. The trend in stabilization is confirmed by the time-dependency of activation energies plotted in Figure 3e.

### Resistance–Time Constant Comparison

Further insights can be drawn from the comparative trends of the time constants (Figure S1) and resistances (Figure S2). As discussed above, *R* is influenced by both the chemical composition and the morphology of the SEI, whereas *τ* is predominantly governed by its chemical properties.

Figure 3f reports the average PCC between the time series of resistances and time constants as a function of temperature, for cells with different electrolyte concentrations. Here, the PCC should be interpreted as the degree to which resistance changes arise from compositional variation (PCC → 1) or morphological evolution (PCC → 0). As clearly shown, the PCC decreases with temperature for the 0.25 m system, remains nearly constant around 0.7 for the 1 m system, and increases towards 1 for the 2 m system. For the 0.25 m electrolyte, this trend suggests that higher temperatures promote increasing morphological evolution of the SEI over time. Since the resistance values *R*_*i*_ tend to increase with time, it is reasonable to infer that the SEI grows in thickness. In contrast, at 1 m, resistance changes appear to be mainly driven by morphological evolution, with limited thickness growth. At 2 m, as the temperature approaches 50 °C, a near-perfect linearity is observed between *R* and *τ*, indicating that the SEI no longer grows appreciably over time.

### XPS and TEM

To further elucidate these observed trends in impedance, complementary SEI chemical characterization was performed using XPS depth profiling. SEI samples were prepared by submerging lithium metal foils in the same LiFSI–G4 electrolyte, and the temperature and salt concentration were varied systematically to uncover the impact these have on SEI chemistry. The F 1s, O 1s, N 1s, C 1s, S 2p, and Li 1s spectra gathered from an SEI sample formed in 1 m LiFSI–G4 for 20 h at 30 °C are presented in Figure 4a. Prior to depth profiling, peaks indicative of LiF, Li_2_SO_4_, N–SO_*x*_ species, and adventitious carbon are detected in the SEI (Table S1), and quantification of the equivalent homogeneous composition in Figure 4b reveals that the sample is carbon-rich. During depth profiling, the adventitious carbon is removed, revealing additional peaks indicative of LiOH, Li_2_O, Li_2_C_2_, and Li_2_S, as well as peaks attributable to organic species containing C–C and C–O bonds. With increasing depth the proportion of carbon detected decreases, while the proportions of oxygen and lithium increase (Figure 4b). These changes are accompanied by the emergence of a Li 1s peak at 52.6 eV, consistent with Li^0^. Notably, fluorine, nitrogen, and sulfur account for less than 5 at. % of all elements detected throughout the SEI, so it is not rich in inorganic, salt-derived species. Carbon also remains prevalent throughout the examined depth.

**Figure 4:**
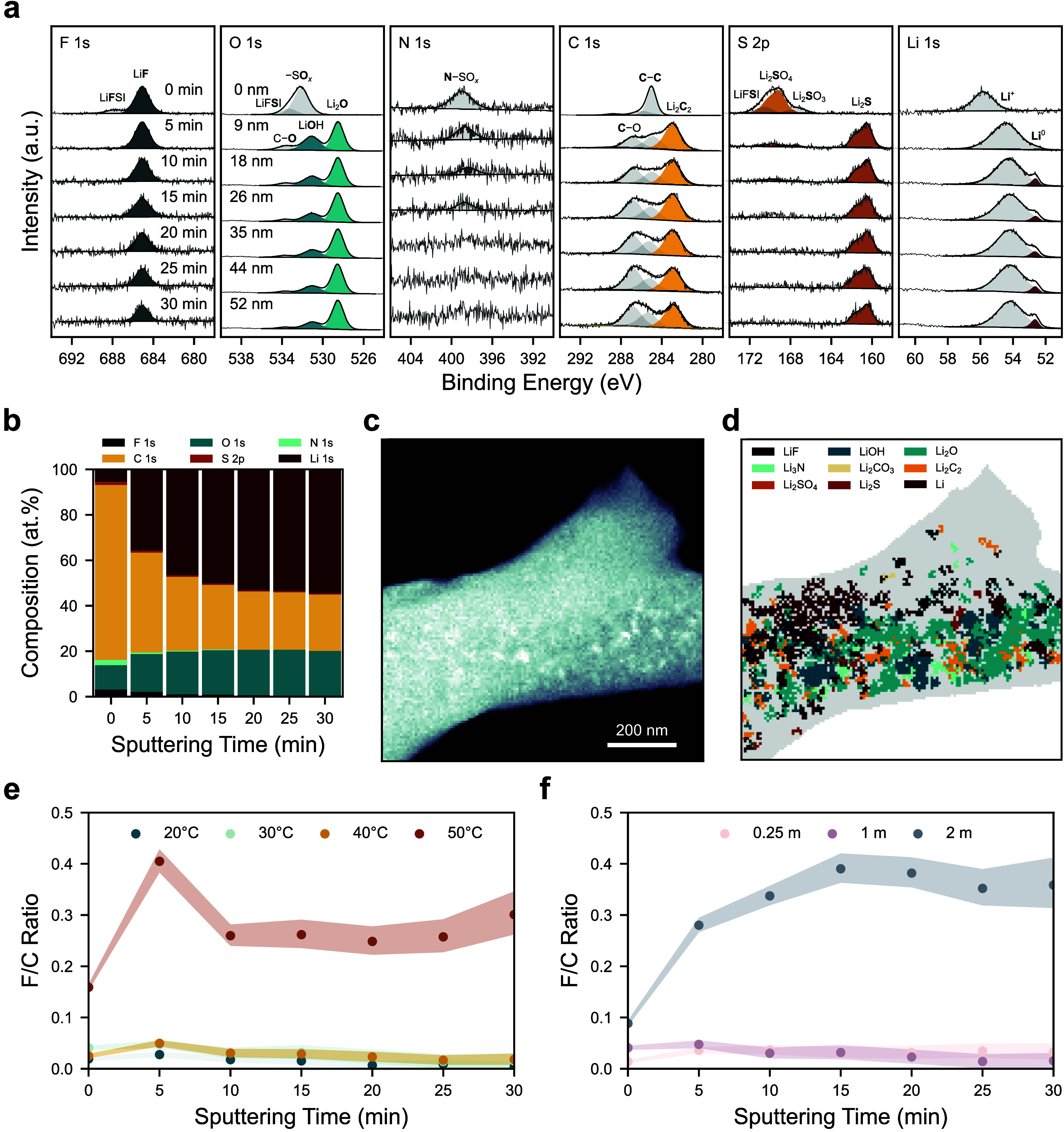
Solid electrolyte interphase chemical characterization. F 1s, O 1s, N 1s, C 1s, S 2p, and Li 1s spectra measured during XPS depth profiling on an SEI sample formed in 1 m LiFSI–G4 for 20 h at 30 °C (a), and equivalent homogeneous composition at each depth (b). Virtual annular dark-field image of a plated lithium metal filament measured with 4D-STEM (c), and map of main crystalline phase detected in each pixel of the sample (d). Depth-dependent fluorine/carbon atomic ratio in the SEI as a function of temperature at 1 m (e), and electrolyte concentration at 30 °C (f). Shaded regions account for uncertainty in peak areas.

It is well-documented that sputter depth profiling can cause sample damage and introduce artefacts in XPS measurements, particularly enhancing Li_2_O concentrations,^35,36^ so low-dose four-dimensional scanning transmission electron microscopy (4D-STEM) imaging was additionally performed on a lithium filament plated in the 1 m LiFSI-G4 electrolyte to confirm the SEI species present. During a 4D-STEM measurement, an electron beam is scanned across the sample, and an electron diffraction pattern is collected at each pixel. Analysis of the recorded diffraction patterns enables the determination of crystalline phases with nanometer spatial resolution.^37^ The virtual annular dark-field (V-ADF) of the filament generated from these diffraction patterns is presented in Figure 4c, and the main crystalline SEI phase that is detected at each pixel of the sample is plotted in Figure 4d. Consistent with the XPS results, Figure 4d suggests that Li_2_O is the main crystalline SEI phase, and LiOH, LiF, Li_2_S and Li_2_C_2_ are also detected.

To determine the impact that temperature has on SEI chemistry, additional XPS measurements were performed on samples formed in 1 m electrolyte at 20, 40, and 50 °C (Figure S3). From 20 to 40 °C there are minimal observed changes in SEI chemistry and it is organic-rich in all three cases, with a fluorine/carbon atomic ratio of approximately 0.02 at all depths (Figure 4e). In contrast, further increasing the temperature to 50 °C results in a significant enhancement in the concentration of inorganic, salt-derived products; the fluorine/carbon ratio in Figure 4e is increased to 0.4 after 5 min of sputtering; and it remains stable at approximately 0.3 with further sputtering. Similar increases in the concentrations of sulfur and oxygen are evident in Figure S4.

Additionally, the impact of electrolyte concentration on SEI chemistry was explored at 30 °C and minimal differences were observed between 0.25 and 1 m (Figure S5), with an organic-rich SEI detected in both cases. Increasing the concentration to 2 m, however, results in an SEI that is markedly richer in fluorine (Figure 4f) and sulfur (Figure S6) compared to carbon.

### Discussion

Taken together, these results provide a coherent picture of the SEI, consistent with the commonly accepted understanding of its structure: a compact, inorganic-rich layer adjacent to the lithium metal, and a porous, organic-rich layer facing the electrolyte.^38,39^

The Arrhenius behaviors observed at 0.25 and 1 m (up to 40 °C) are indicative of a uniform SEI composition, while the activation energies in the range of 0.25–0.44 eV are comparable to the value reported for lithium diffusion in 1 m LiFSI–G4 (0.23 eV) in a previous study by our group.^25^ This suggests that, in these conditions, ion transport is primarily governed by transport in the organic–porous layer through the liquid electrolyte occupying the pores.

The change in SEI composition at a 1 m concentration upon increasing the temperature to 50 °C can be rationalized by considering the temperature dependence of solvation structures in a 1 m LiFSI–G4 electrolyte, as explored by Olbrich et al.^25^ At 20 °C approximately 50% of the FSI^–^ is “free” while the rest is “bound”, denoting FSI^–^ that is not associated with the Li^+^ and FSI^–^ that is in the primary solvation shell of the Li^+^, respectively. This leads to a solvation number (average number of ions/molecules surrounding the central ion) of approximately 0.5, while the solvation number of G4 is 1.3.^23^ SEI formation is believed to occur by the reduction of species in the primary solvation shell of the cation, so the high concentration of G4 results in the organic-rich SEI observed at lower temperatures. The proportion of bound FSI^–^ increases with increasing temperature due to an increase in entropy associated with the exchange of solvent molecules for FSI^–^ in the solvation shell,^40^ reaching 59% at 50 °C,^25^ so there will be more FSI^–^ available for reduction. Combining this with the higher reactivity at higher temperatures appears to result in the anion-derived SEI observed here. These trends in SEI chemistry are consistent with the temperature-dependent impedance results at 1 m and suggest that an inorganic, anion-derived SEI is more ionically conductive in this system.

Increasing concentration to 2 m concentration also leads to changes in composition. EIS analysis reveals faster time constants and an increase in activation energies (0.6 eV), aligning with trends observed for solid-state conduction in dense SEIs,^41^ thus suggesting the presence of a greater proportion of inorganic components in the SEI. This is corroborated by XPS analysis shown in Figure S5 for samples prepared at 30 °C. Compared with samples prepared at the same temperature but lower concentrations, the SEI formed at 2 m is composed of more inorganic species. This shift in composition is likely caused by alterations in the solvation shell surrounding Li^+^, which evolves as the electrolyte concentration exceeds 1 m, as evidenced by the sharp rise in the thermodynamic factor reported by Fawdon et al.^23^ This inorganic, compact SEI forms rapidly and provides strong passivation, suppressing further electron transfer and electrolyte diffusion, thereby halting growth as supported by our resistance–time analysis.

It is worth noting that the SEI formed at 1 m and 50 °C and that formed at 2 m across all temperatures are comparable when corrected for bulk electrolyte resistance, as observed by the respective impedance spectra (Figure S7). This suggests a similar composition and, potentially, similarities in the bulk solvation structure.

## Conclusions

In this study, we present an extensive EIS investigation of lithium metal in the model electrolyte LiFSI–G4. The system is analyzed at equilibrium as a function of time, temperature, and salt concentration, using a custom-designed setup to minimize measurement artifacts. We apply statistical methods to determine the number of independent degrees of freedom and to constrain the number of Voigt elements used in the fitting process.

Our results reproduce SEI composition trends consistent with the literature, namely, a compact, inorganic-rich layer adjacent to lithium metal and a more porous, organic-rich layer toward the electrolyte. This validates our experimental design and, more importantly, demonstrates the robustness of our information-theory-guided fitting approach. Furthermore, we show that variations in temperature and concentration can promote the formation of a more inorganic-rich SEI.

We observe strong correlations among the different Voigt elements used to fit the EIS spectra. All time constants exhibit similar trends across experimental conditions, suggesting that they reflect a single, distributed dominant process rather than discrete physical layers or mechanisms. We therefore advise caution when assigning individual physical meaning to each circuit element in such fits, as is frequently done in literature. Importantly, this distribution cannot be captured by a simple R/CPE-type element, given the system’s complexity, as further demonstrated by the statistical algorithm presented in this work.

We further demonstrate that independent and complementary analysis of time constants and resistances enables the decoupling of compositional and morphological changes in the SEI. The trend of average activation energy across *τ*_*i*_ vs salt concentration shows good agreement with composition trends identified by XPS, whereas resistance trends do not. This discrepancy likely arises because resistances, whether obtained from fits or extracted from the geometrical analysis of the spectra, are influenced by morphological factors such as layer thickness and porosity, which cannot be disentangled from composition alone.

We therefore emphasize that, assuming appropriate cell design, a reliable interpretation of the system requires both equivalent circuit fitting and time-constant analysis. While parameters extracted directly from the spectra—such as total resistance—are objective and reproducible, they are insufficient to isolate compositional effects without additional analysis.

This study highlights the potential of using equilibrium EIS analysis to identify optimal combinations of temperature and time during initial SEI formation, enabling the design of favorable SEI compositions for lithium metal anodes without relying on highly concentrated electrolytes. While the SEI will be influenced by subsequent plating and stripping cycles, the initial SEI has been shown to be one of the main determining factors governing cycling performance.^42^ Decoupling electrolyte concentration from initial SEI formation could therefore help avoid the cost and transport penalties associated with highly concentrated electrolytes.

Finally, we would like to highlight the unique and extensive dataset we have collected, comprising over 12000 highly reproducible impedance spectra measured as a function of time, temperature, and concentration, with three independent repeats for each condition. This dataset, made possible by meticulous cell and experimental design, is available to the reader. We are confident that it will serve as a valuable resource for further analysis, as well as for supporting future modeling and experimental efforts.

## Supplementary Material



## Notes

Experimental details for impedance and 4D-STEM measurements; time series for time constants and resistances; XPS analysis results and peak fitting parameters; additional impedance-related plots (PDF)
