# Supplementary material for: Electrochemical Impedance Spectroscopy Investigation of the SEI Formed on Lithium Metal Anodes
Source: ACS Electrochem. 2025 Nov 24;2(1):166–74. doi: 10.1021/acselectrochem.5c00388 (PMC12766685; doi:10.1021/acselectrochem.5c00388)
Supplement: Supplementary file 1 [file ec5c00388_si_001.pdf]

Supporting Information
Electrochemical Impedance Spectroscopy Investigation of the SEI Formed on Lithium Metal Anodes

Lorenz F. Olbrich,[†,⊥] Nicolò Pianta,[‡,†,⊥] Ben Jagger,[†] Yiming Xu,[†] Manav Kakkanat,[†] Federico Scarpioni,[¶] Christopher Allen,[†,§] Fabio La Mantia,[‖,¶] Riccardo Ruffo,[‡] and Mauro Pasta[*,†]

†*Department of Materials, University of Oxford, Oxford OX1 3PH, United Kingdom*

‡*Department of Materials Science, University Milano - Bicocca, Via Roberto Cozzi 55, Milan (MI), 20125, Italy*

¶*Fraunhofer Institute for Manufacturing Technology and Advanced Materials IFAM, Wiener Strasse 12, Bremen 28359, Germany*

§*Electron Physical Science Imaging Centre, Diamond Light Source Ltd., Didcot OX11 0DE, United Kingdom*

‖*Universität Bremen, Energiespeicher-und Energiewandlersysteme, Bibliothekstr. 1, Bremen 28359, Germany*

⊥*These authors contributed equally.*

E-mail: mauro.pasta@materials.ox.ac.uk

Contents

Experimental Methods **S3**

Electrolyte preparation S3

Electrode preparation S3

Cell assembly S3

Electrochemical Impedance Spectroscopy S4

Impedance fitting S5

Quality of impedance data and fitting S5

X-ray Photoelectron Spectroscopy S6

4D-STEM S7

Sample preparation S7

TEM grid preparation S7

4D-STEM acquisition S7

Data analysis S8

Additional Table and Figures **S9**

References **S16**

Experimental Methods

Unless otherwise stated, all sample preparation was conducted in argon-filled MBraun gloveboxes, with O_2 and H_2O concentrations maintained below 0.1 ppm. All equipment was cleaned and dried under vacuum at 70 °C overnight prior to being transferred into the glovebox.

Electrolyte preparation

Prior to electrolyte preparation, lithium bis(fluorosulfonyl)imide (LiFSI, battery grade, Fluorochem) was dried under vacuum at 70 °C for a minimum of 48 hours, and tetraethylene glycol dimethyl ether (G4, 99%+, Sigma-Aldrich) was dried over 3 Å molecular sieves. Electrolytes were prepared gravimetrically by stirring the desired masses of salt and solvent. The water content of the resulting electrolytes was measured by Karl Fischer titration and found to be below 5 ppm. Three concentrations were prepared for investigation in this work: 0.25, 1 and 2 molal (moles of salt per kilogram of solvent).

Electrode preparation

Lithium metal electrodes were prepared by first brushing the surface of lithium (99.9% trace metal basis, Sigma-Aldrich) with a plastic brush, followed by calendering to a thickness of 300 µm inside a polypropylene bag. The lithium was then punched into 8 mm diameter disks using a wad punch.

Cell assembly

To collect impedance spectra with minimal artifacts—that is, to reduce distortions arising from third-electrode positioning, separator effects, and imperfect contact between the electrolyte and current collectors—a custom two-electrode cell was employed. In this configuration, both electrodes and their interphases are identical and symmetrically aligned.

Consequently, the interphases are connected in series, and the impedance contribution of a single interface can be obtained by dividing the measured impedance by two. This assumption is valid under open-circuit voltage (OCV) conditions, provided that electrode symmetry is maintained. The cell consists of a hollow cylinder with an internal diameter of 8 mm, onto which two stainless steel current collectors are screwed, separated by a distance of 1 mm. Lithium was affixed to the current collectors by simple mechanical pressure. The lithium surface was cleaned by vigorous scrubbing with a dedicated toothbrush. To minimise the influence of surface roughness, the lithium was pressed onto the current collector using a hydraulic press at 50 MPa. A drop of hexane was applied between the lithium and the pressing tool to prevent sticking. Excess lithium, which overflowed during pressing, was carefully removed using a razor blade. The entire inner chamber of the cell was then filled with electrolyte, ensuring that no gas was trapped during closure.

For the comparison shown in Figure 1, a coin cell (2032 geometry, MTI Corporation) was assembled. Lithium metal, prepared in the same manner as for the custom cell, was used for both electrodes. A volume of 150 µL of the 1 m solution was added along with a glass fiber separator (Whatman, GF/D).

Electrochemical Impedance Spectroscopy

Impedance spectra were collected using a VSP-300 Biologic potentiostat/galvanostat over a frequency range of 200 kHz to 100 mHz, with 6 points per decade and an excitation amplitude of 10 mV. Measurements were taken every 2 min over a total duration of 10 h. To optimize measurement time, data acquisition began immediately after cell assembly. To avoid artifacts related to thermal equilibration, the spectra collected during the first 20 min were excluded from the analysis. For each temperature–electrolyte concentration pair, a minimum of three cells were assembled and tested to ensure reproducibility of the method.

Impedance fitting

All impedance spectra were fitted using a custom-modified branch of the *pymultipleis* Python library (`https://pypi.org/project/pymultipleis/`), employing nonlinear least squares minimization with the "adam" stochastic solver. Unlike conventional fitting software, the approach used in this work allows the simultaneous treatment of an entire dataset (i.e., all spectra from a single cell), enforcing temporal smoothness in the evolution of fitting parameters through a smoothing factor that penalizes abrupt changes in their second derivatives with respect to time. For clarity, all quantities, parameters, and values derived from the impedance analysis are reported as statistical means across all cells measured under identical temperature and electrolyte concentration conditions. Reported uncertainties for the fitting parameters correspond to the total propagated uncertainty, calculated using standard error propagation methods.

Quality of impedance data and fitting

The quality of the impedance data and the corresponding fits was assessed through evaluation of the reduced chi-squared statistic, χ^2:

$$\chi^2 = \frac{1}{2n_f - n_\theta} \left|\left| \frac{Z - Z_{fit}}{|Z|} \right|\right|^2 \tag{1}$$

where n_f and n_θ represent the number of frequency points and model parameters, respectively; Z denotes the measured impedance data, and Z_{fit} the corresponding fitted values. This expression represents the weighted residual norm of the fit.

Fitting with the model presented in Figure 2 yields an average χ^2 of $5(1) \times 10^{-6}$, corresponding to an AIC of $-500(100)$—values that highlight the quality of the fit.

When fitting the transfer function of order n, we were also able to assess the validity of the spectra with respect to the Kramers–Kronig (KK) transformations. Notably, the fitting residuals are consistently below 0.5% when $n > 7$. This is expected, as equation 1

intrinsically satisfies the KK relations, meaning that any data well fitted by this model naturally complies with them. This approach is analogous to methods that use a series of Voigt elements to fit impedance spectra (see, for example,[1]).

X-ray Photoelectron Spectroscopy

SEI samples for X-ray photoelectron spectroscopy (XPS) were prepared by pressing lithium metal disks onto a stainless steel spacer (304SS, MTI) and submerging this in 1 mL of electrolyte. These were then kept in a glovebox antechamber heated to the stated temperature (30–50°C), except the sample formed at 20°C was placed in a Binder Oven. The samples were kept in electrolyte for the stated time (20 h) before they were removed from the electrolyte and rinsed three times each with 200 µL of pure solvent to remove residual salt. The excess solvent was then removed with tissue, taking care not to touch the electrode surface. Samples were then immediately transferred to the XPS intro chamber using a vacuum transfer vessel (ULVAC PHI) to prevent air exposure.

XPS was performed with an ULVAC PHI Versaprobe III XPS system generating monochromatic $Al_{K\alpha}$ X-rays (1486.6 eV, 15 kV anode voltage, 25 W beam power) under ultrahigh vacuum (UHV) conditions (10^{-7}–10^{-6} Pa). A 500 µm × 500 µm area of each sample was analysed. Core-level spectra were gathered at a pass energy of 55 eV. In-built electron and low-energy Ar^+ sources were utilized for neutralization. Depth profiling was achieved with consecutive XPS analysis and Ar^+ sputtering (2 kV, 3 mm × 3 mm). Acquired spectra were fitted with Gaussian-Lorentzian product lineshapes (with an asymmetric lineshape necessary to fit the Li^0 Li 1s peak) using CasaXPS software.[2] Spectra were charge referenced to the LiF peak at 685.1 eV.[3] Atomic percentages were estimated using relative sensitivity factors determined from LiFSI (battery grade, Fluorochem), Li_2O (99.5%, Thermo Fisher) and LiOH (98%, Sigma-Aldrich). The sputtering rate was measured to be 1.75 nm min^{-1} for a Li_2O standard.

4D-STEM

Sample preparation

Lithium filaments were prepared using a custom Hittorf-like cell filled with the electrolyte of choice. Immediately after assembly, the cell was rested for 1.5 hours to allow the temperature to equilibrate to 20 °C. Plating was then carried out at a current density of 600 µA cm^{-2} for 9 hours. All subsequent handling steps were performed in an argon-filled glovebox, with O_2 and H_2O concentrations maintained below 0.1 ppm. The harvested filaments were stored in a suspension of the native electrolyte to preserve their native SEI structures.

TEM grid preparation

The filaments were loaded onto the TEM grid immediately prior to the imaging session. Room-temperature 4D-STEM acquisitions were performed using a JEOL double-tilt vacuum transfer holder. The TEM grid was mounted onto the holder inside an argon-filled glovebox. After insertion into the TEM column, the specimen chamber was evacuated and purged with argon three times before opening the vacuum transfer holder, in order to minimise exposure to air.

4D-STEM acquisition

4D-STEM acquisitions were performed at 300 kV using a JEOL ARM300CF at the Electron Physical Science Imaging Centre (ePSIC) at Diamond Light Source (United Kingdom). The near-parallel nanobeam 4D-STEM mode employed a probe diameter of 3 nm and a convergence angle of 1 mrad. The real-space scanning step size ranged from 2–4 nm to maximize the efficiency of electron fluence for information acquisition. The beam current (0.2 pA) was measured using a Faraday cup positioned inside the TEM column. Electron diffraction patterns (DPs) were recorded using a pixelated MerlinEM 4R direct electron detector. Unless otherwise specified, 4D-STEM datasets were acquired using a camera length of 40 cm and a

dwell time of 600 µs. The average electron dose per acquisition was $3.07\,e^{-}\,\text{Å}^{-2}$. Real-space 4D-STEM data (initially acquired at 256×256 scan pixels) were binned to 128×128 during data processing.

Data analysis

The 4D-STEM (nanobeam) data were analyzed as follows. First, the two-dimensional diffraction data were converted into one-dimensional radial intensity profiles. These profiles, concatenated across multiple scan areas, were subjected to non-negative matrix factorization (NMF) to enable real-space clustering. The diffraction pattern (DP) intensities were then summed within each real-space cluster, significantly improving the signal-to-noise ratio. Bragg reflections were identified on the resulting clustered DPs and subsequently indexed to assign the most plausible crystalline phases present within the scanned regions.

- py4DSTEM version: 0.14.17
- hyperspy version: 2.2.0
- h5py version: 3.9.0
- pandas version: 1.5.3
- scipy version: 1.12.0
- matplotlib version: 3.9.1
- scikit-image version: 0.22.0
- numpy version: 1.26.4
- python version: 3.10.13 | packaged by conda-forge | (main, Dec 23 2023, 15:36:39) [GCC 12.3.0]

Additional Table and Figures

Table S1: XPS fitting parameters and peak assignments corresponding to Figure 4a.

Transition	Species	Binding Energy (eV)	FWHM (eV)	Spin-Orbit Splitting (eV)	References
F 1s	LiFSI	687.9	1.6	–	[3,4]
	LiF	685.1	1.6	–	[3]
O 1s	LiFSI	533.3	1.7	–	[4]
	$-SO_x$	532.1	1.7	–	[4,5]
	C−O	533.7	1.5	–	[5]
	LiOH	531.1	1.9	–	[3,6]
	Li_2O	528.5	1.3	–	[6]
N 1s	$N-SO_x$	$\sim$ 399	2.0	–	[4]
C 1s	C−O	286.9	1.9	–	[5]
	C−C	284.9	1.9	–	[5]
	Li_2C_2	282.9	1.9	–	[7]
S 2p	LiFSI	170.4	1.5	1.16	[4]
	Li_2SO_4	169.1	1.5	1.16	[8,9]
	Li_2SO_3	167.0	1.5	1.16	[8,9]
	Li_2S	160.5	1.2	1.16	[4,8]
Li 1s	LiFSI/LiF/Li_2SO_x	55.8	1.5	–	[4]
	Li_2O	54.5	1.2	–	[6,10]
	Li^0	52.6	0.5	–	[6,10]

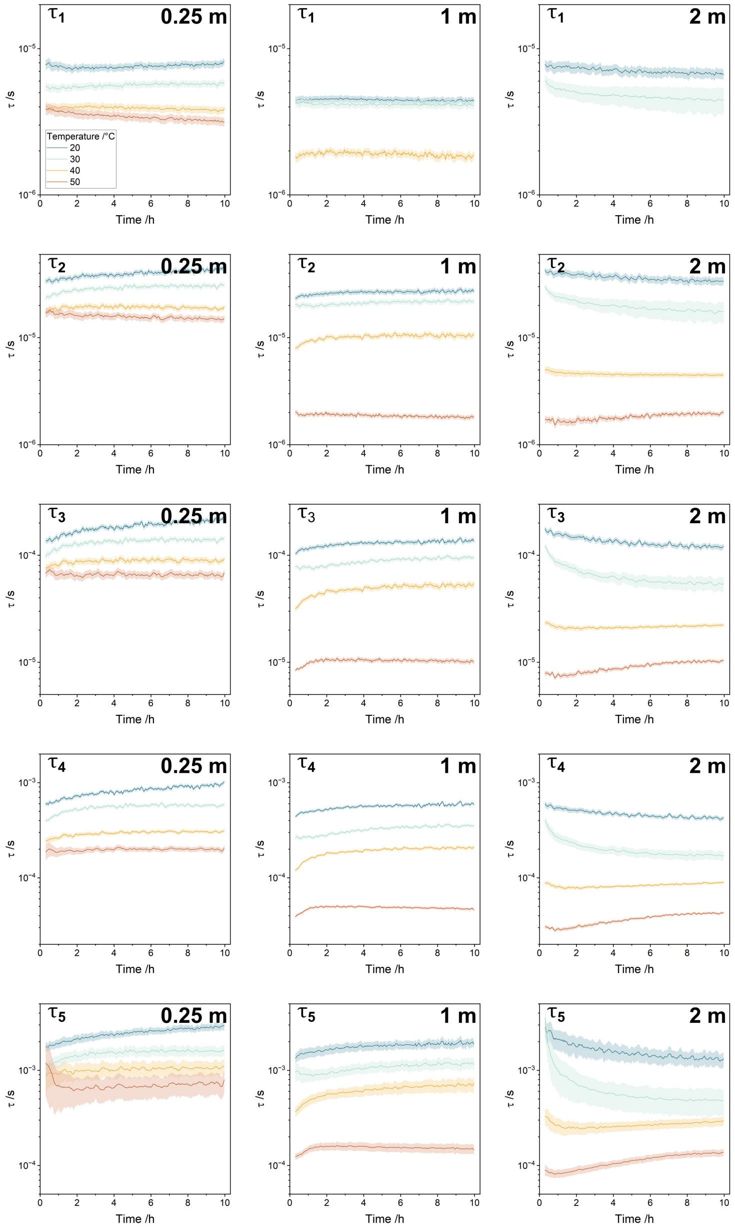

Fig. S1: **Time series of all the time constants:** Time constant vs time for all the τ_i obtained by fitting the impedance dataset using the multilayer model.

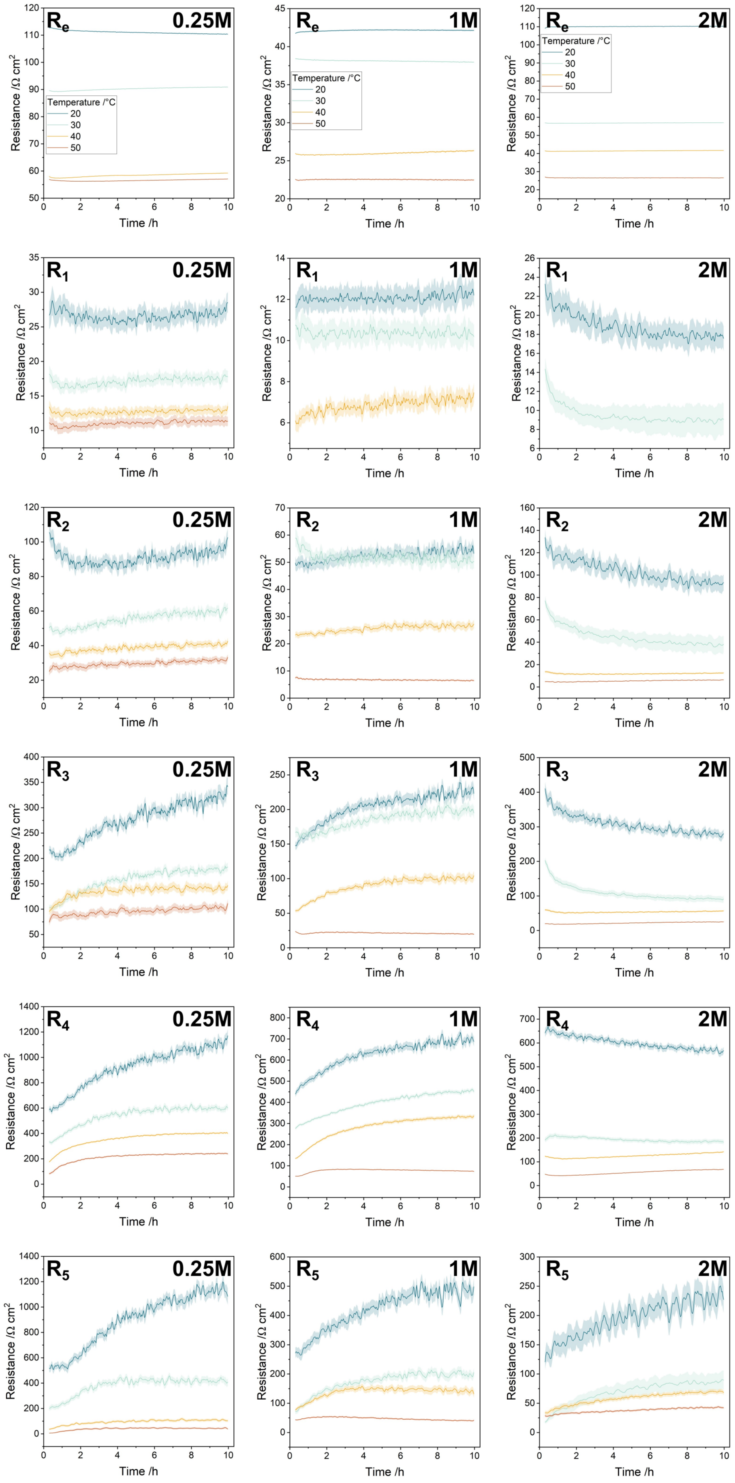

Fig. S2: **Time series of all the resistances:** Resistance vs time for all the resistances obtained by fitting the impedance dataset using the multilayer model.

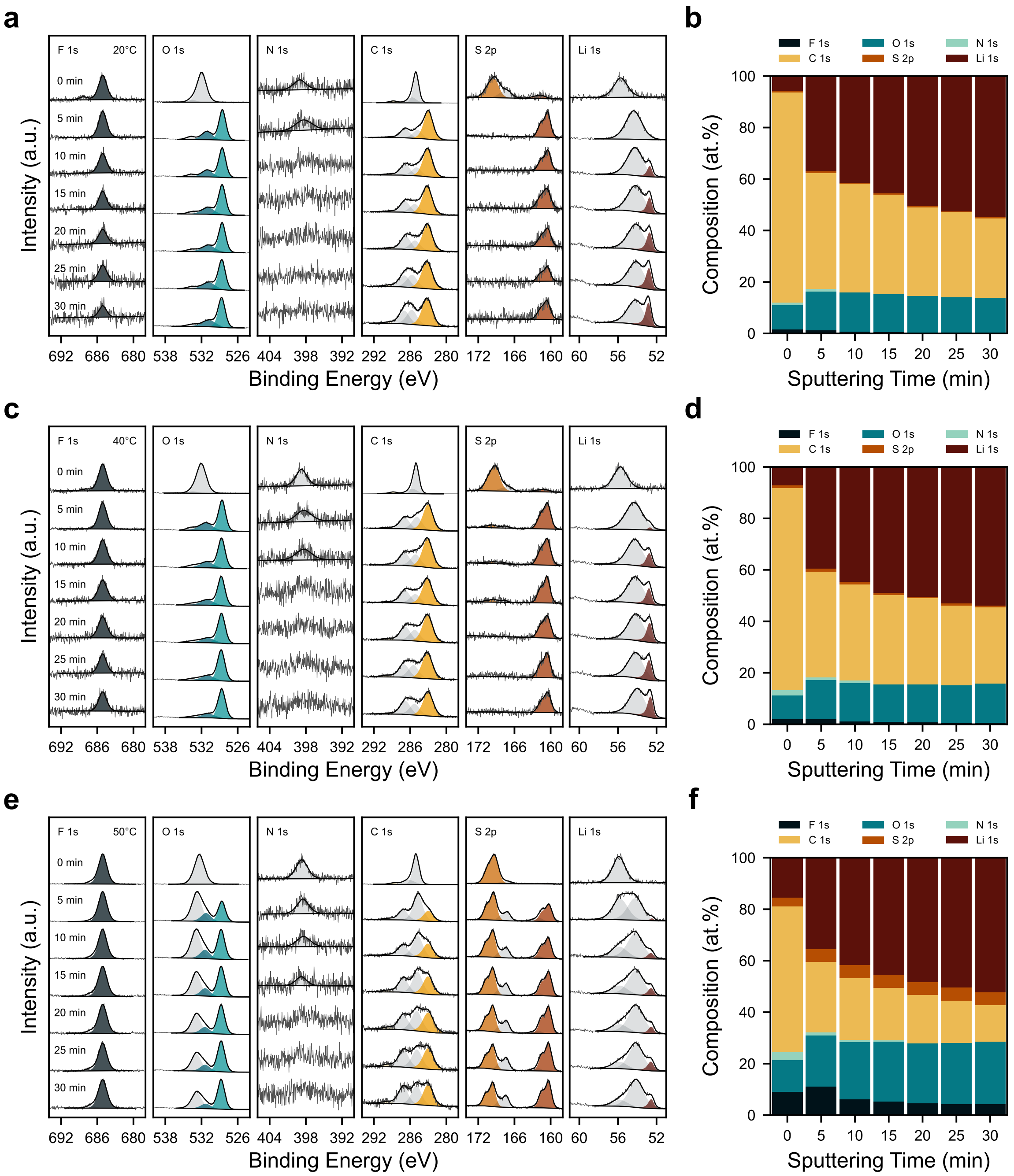

Fig. S3: F 1s, O 1s, N 1s, C 1s, S 2p and Li 1s spectra measured during XPS depth profiling on SEI samples formed in 1 m LiFSI–G4 for 20 h at various temperatures (**a, c, e**), and equivalent homogeneous composition at each depth (**b, d, f**). 20 °C (**a, b**), 40 °C (**c, d**), 50 °C (**e, f**).

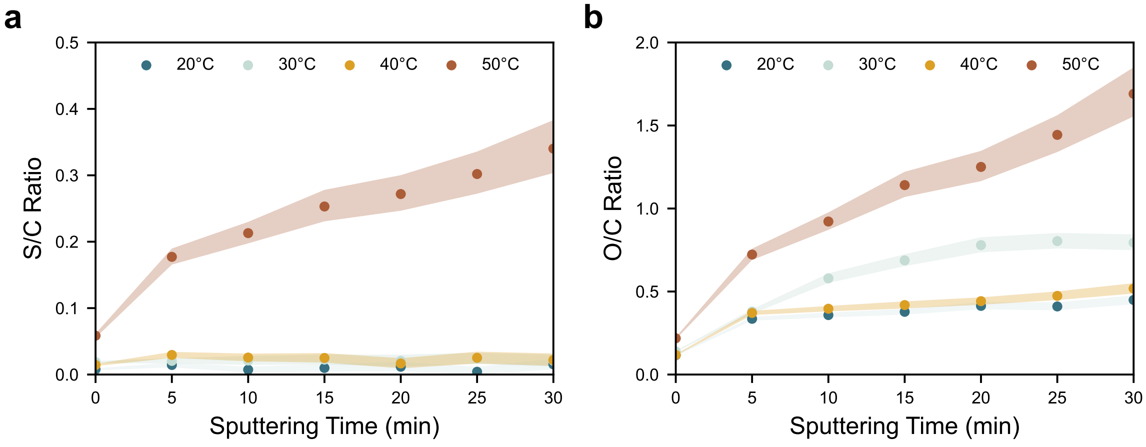

Fig. S4: Depth-dependent sulfur/carbon (**a**) and oxygen/carbon (**b**) atomic ratios in the SEI as a function of temperature at 1 m. Shaded regions account for uncertainty in peak areas.

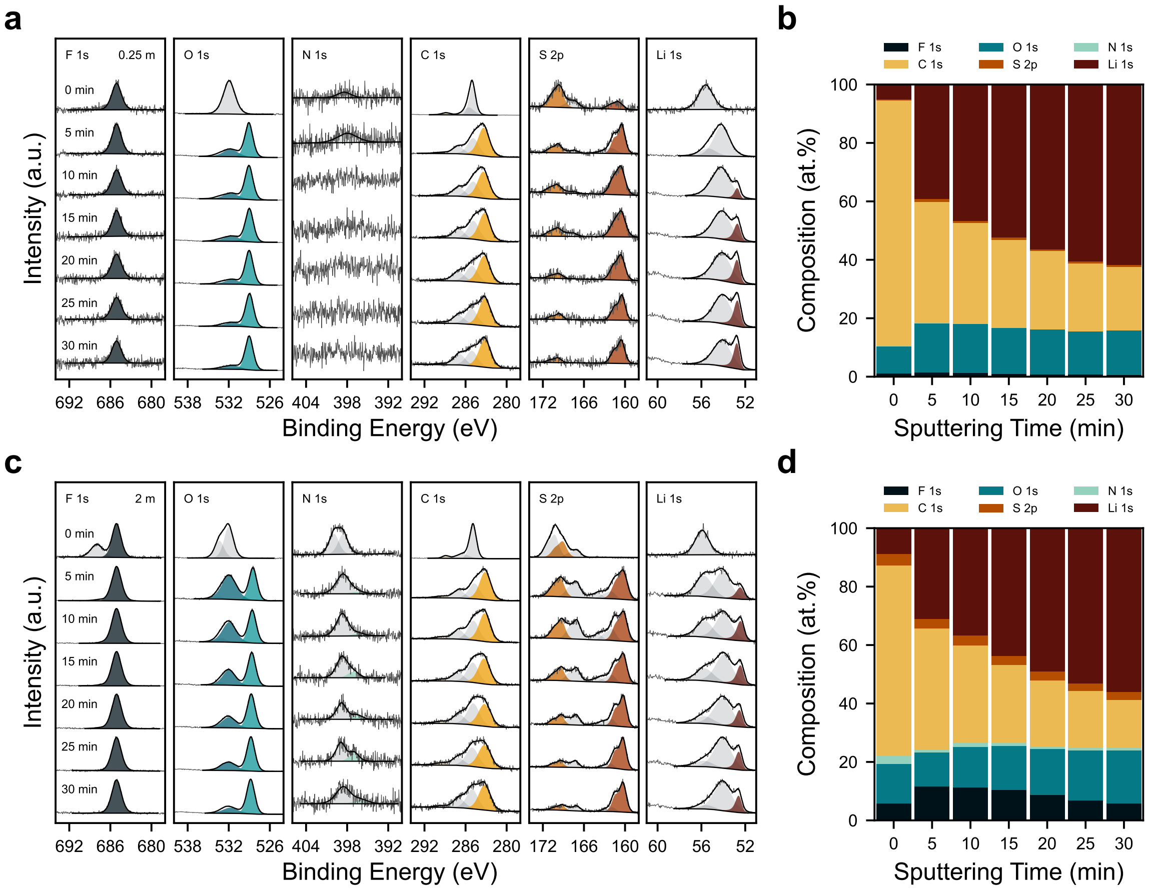

Fig. S5: F 1s, O 1s, N 1s, C 1s, S 2p and Li 1s spectra measured during XPS depth profiling on SEI samples formed in LiFSI–G4 electrolytes of various concentrations for 20 h at 30 °C (**a, c**), and equivalent homogeneous composition at each depth (**b, d**). 0.25 m (**a, b**), 2 m (**c, d**).

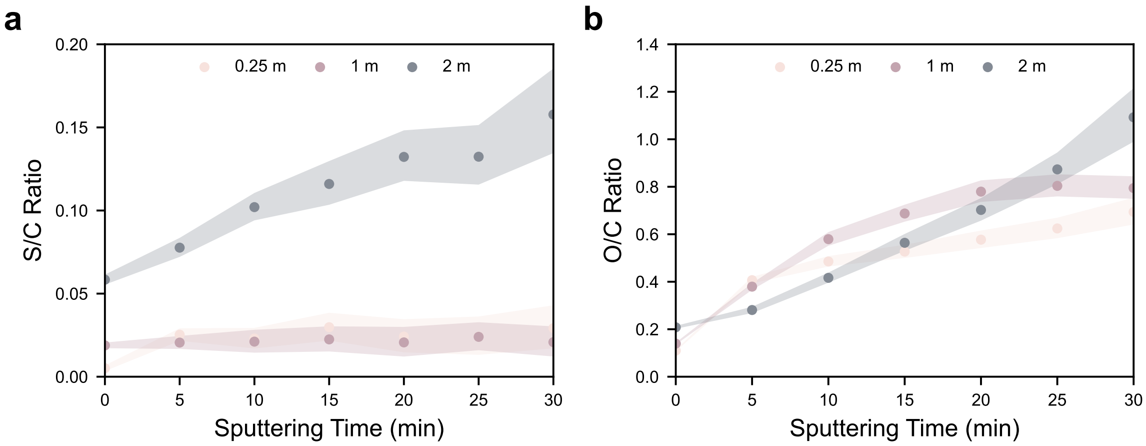

Fig. S6: Depth-dependent sulfur/carbon (**a**) and oxygen/carbon (**b**) atomic ratios in the SEI as a function of concentration at 30 °C. Shaded regions account for uncertainty in peak areas.

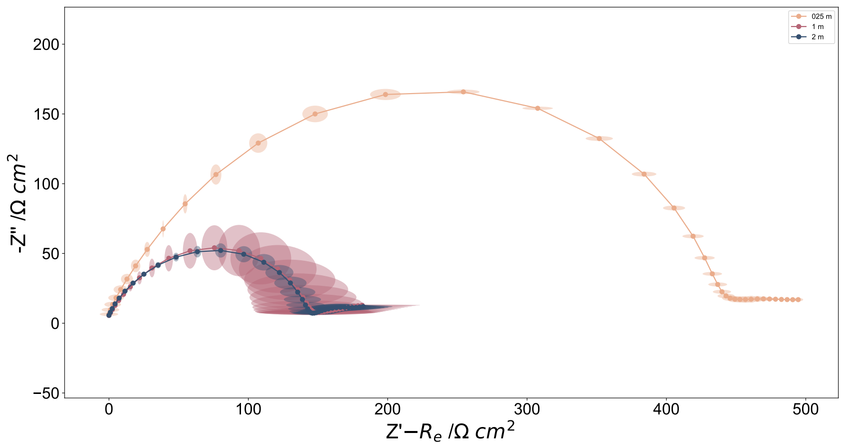

Fig. S7: **Electrolyte's resistance-corrected impedance spectra:** Comparison of the impedance spectra, averaged across the last hour of measurement at 50 °C for the three electrolyte's composition. The error of each impedance point is expressed as an ellipse around the point.

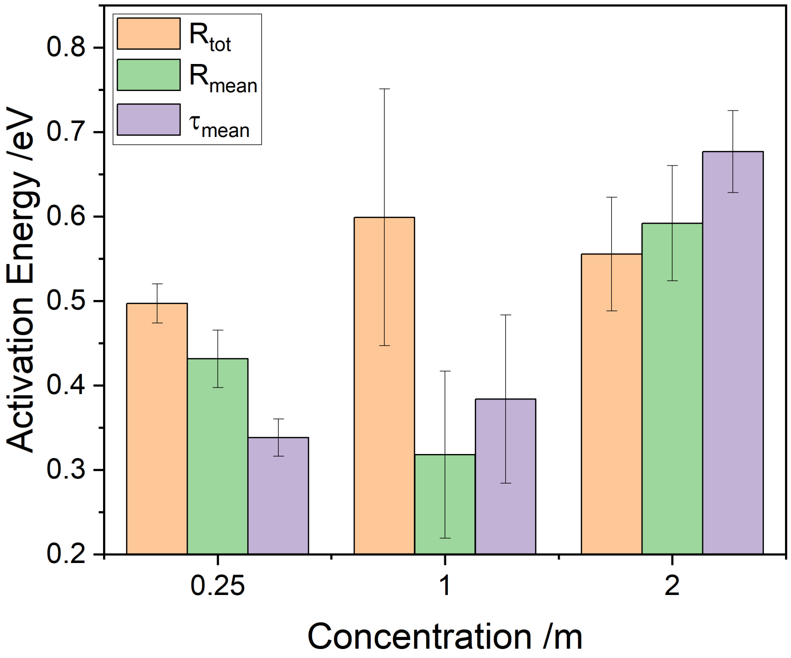

Fig. S8: **Resistance vs. time constants:** Comparison of activation energies associated with total resistance ($R_{\rm tot}$), mean resistance from fitting ($R_{\rm mean}$), and mean time constant ($\tau_{\rm mean}$), as a function of electrolyte concentration. Only $\tau_{\rm mean}$ follows the trend observed in the XPS analysis.

References

(1) Kauffman, G. B. Electrochemical Impedance Spectroscopy. By Mark E. Orazem and Bernard Tribollet. *Angewandte Chemie International Edition* **2009**, *48*.

(2) Fairley, N.; Fernandez, V.; Richard-Plouet, M.; Guillot-Deudon, C.; Walton, J.; Smith, E.; Flahaut, D.; Greiner, M.; Biesinger, M.; Tougaard, S.; Morgan, D.; Baltrusaitis, J. Systematic and collaborative approach to problem solving using X-ray photoelectron spectroscopy. *Applied Surface Science Advances* **2021**, *5*, 100112.

(3) Caracciolo, L.; Madec, L.; Martinez, H. XPS Analysis of K-based Reference Compounds to Allow Reliable Studies of Solid Electrolyte Interphase in K-Ion Batteries. *ACS Appl. Energy Mater.* **2021**, *4*, 11693–11699.

(4) Yu, W. et al. Electrochemical Formation of Bis(fluorosulfonyl)imide-Derived Solid-Electrolyte Interphase at Li-Metal Potential. *Nat. Chem.* **2025**, *17*, 246–255.

(5) Henderson, J. ; Payne, B. ; McIntyre, N. ; Biesinger, M. Enhancing Oxygen Spectra Interpretation by Calculating Oxygen Linked to Adventitious Carbon. *Surf. Interface Anal.* **2025**, *57*, 214–220.

(6) Otto, S. K.; Moryson, Y.; Krauskopf, T.; Peppler, K.; Sann, J.; Janek, J.; Henss, A. In-Depth Characterization of Lithium-Metal Surfaces with XPS and ToF-SIMS: Toward Better Understanding of the Passivation Layer. *Chem. Mater.* **2021**, *33*, 859–867.

(7) Kanamura, K.; Shiraishi, S.; Tamura, H.; Takehara, Z. X-Ray Photoelectron Spectroscopic Analysis and Scanning Electron Microscopic Observation of the Lithium Surface Immersed in Nonaqueous Solvents. *J. Electrochem. Soc.* **1994**, *141*, 2379–2385.

(8) Lacey, M. J.; Yalamanchili, A.; Maibach, J.; Tengstedt, C.; Edström, K.; Brandell, D. The Li–S battery: An Investigation of Redox Shuttle and Self-Discharge Behaviour with $LiNO_3$-Containing Electrolytes. *RSC Adv.* **2016**, *6*, 3632–3641.

(9) Fujita, Y.; Motohashi, K.; Ding, J.; Tsukasaki, H.; Mori, S.; Sakuda, A.; Hayashi, A. Lithium Sulfite Enhances Cycle Performance of All-Solid-State Batteries with Li_2S-Based Positive Electrode Materials. *ACS Appl. Energy Mater.* **2024**, *7*, 5447–5456.

(10) Wood, K. N.; Teeter, G. XPS on Li-Battery-Related Compounds: Analysis of Inorganic SEI Phases and a Methodology for Charge Correction. *ACS Appl. Energy Mater.* **2018**, *1*, 4493–4504.
